# *Drosophila melanogaster* as a Model Organism to Study Lithium and Boron Bioactivity

**DOI:** 10.3390/ijms222111710

**Published:** 2021-10-28

**Authors:** Katharina Jans, Kai Lüersen, Gerald Rimbach

**Affiliations:** Institute of Human Nutrition and Food Science, University of Kiel, 24118 Kiel, Germany; luersen@foodsci.uni-kiel.de (K.L.); rimbach@foodsci.uni-kiel.de (G.R.)

**Keywords:** lithium, boron, trace elements, fruit fly, model organism, longevity, health span

## Abstract

The fruit fly *Drosophila melanogaster* has become a valuable model organism in nutritional science, which can be applied to elucidate the physiology and the biological function of nutrients, including trace elements. Importantly, the application of chemically defined diets enables the supply of trace elements for nutritional studies under highly standardized dietary conditions. Thus, the bioavailability and bioactivity of trace elements can be systematically monitored in *D. melanogaster*. Numerous studies have already revealed that central aspects of trace element homeostasis are evolutionary conserved among the fruit fly and mammalian species. While there is sufficient evidence of vital functions of boron (B) in plants, there is also evidence regarding its bioactivity in animals and humans. Lithium (Li) is well known for its role in the therapy of bipolar disorder. Furthermore, recent findings suggest beneficial effects of Li regarding neuroprotection as well as healthy ageing and longevity in *D. melanogaster*. However, no specific essential function in the animal kingdom has been found for either of the two elements so far. Here, we summarize the current knowledge of Li and B bioactivity in *D. melanogaster* in the context of health and disease prevention.

## 1. Introduction

### 1.1. Categorization and Functionality of Trace Elements

Along with vitamins, bulk elements, and macrominerals, trace elements (TE) belong to the group of micronutrients. They differ from the energy- and structure-providing macronutrients, as they are required in rather low quantities while still playing a vital role in maintaining physiological functions and metabolic processes [1]. TE usually hold essential functions as catalytics, co-factors, or as structural components of larger molecules, called metallo-proteins and metallo-enzymes, while they only require intake levels within the µg–mg range and make up less than 0.01% of an organism’s mass [1,2,3,4,5]. As essential TE exert either one specific or, quite commonly, even more vital functions (called metabolic multi-functionality), it is crucial for the organism to obtain a physiological amount within a range that allows maintenance of its actions [2]. While intake levels of nutrients naturally vary from time to time, dependent upon given food sources and other external factors, the organism has to cope with a rather fluctuating supply [6]. Therefore, there are a number of evolutionary conserved homeostatic mechanisms to regulate absorption, distribution, storage, and elimination in order to avert both deficiency and intoxication [7].

In 1996, the World Health Organization (WHO) published the outcome of the Joint Expert Consultation on Trace Elements in Human Nutrition, whereby 19 TE were further categorized into three groups: “essential”, “probably essential (including boron, B)”, and “potentially toxic, some possibly with essential function (including lithium, Li)” [8].

B and Li are two promising candidate elements that have been in the center of interest to elucidate biological actions and potentially vital functions in higher organisms for decades. However, whether or not B and Li hold general evolutionary conserved essential functions in the animal kingdom has yet to be investigated. In order to provide better understanding of both elements, we gathered the current knowledge on B and Li that was yielded from the fruit fly while assessing the value and suitability of the *Drosophila*-model in the study of both elements, respectively.

### 1.2. The Model of D. melanogaster in Trace Element Homeostasis

The fruit fly has a long history as a powerful model in biomedical research. While it is an easy-to-maintain and low-cost organism, the fruit fly allows high-throughput analyses due to its high reproductive rate and a rather short lifespan [9]. Since *D. melanogaster* has been used as a genetic model in the first place, the fruit fly research community has created a capable toolbox that offers a broad spectrum of forward and reverse genetics methodology [10]. Carefully applied, highly evolutionary conserved pathways and functional homology with the human genome indicate the value of *Drosophila*-gathered research also for human nutrition and pharmacology [11]. Accordingly, the model of *D. melanogaster* has since been versatilely applied to study biological actions and toxicity of chemical elements [9].

Up to now, six TE were found to display clear signs of essentiality in the fruit fly, namely bromine, copper, iron, manganese, molybdenum, and zinc. All six have been demonstrated to perform a defined biological function required to complete the lifecycle and/or display other symptoms of deficiency [12,13,14,15,16,17,18,19,20]. It should be noted here that bromine was first found to be essential as a cofactor in collagen synthesis using the model of *D. melanogaster* emphasizing the power of the fruit fly model in TE research. In the fly, bromine has proven to be required during development since a bromine-free diet results in a significantly reduced hatching rate relative to bromine supplementation. Essentiality of bromine was fully confirmed, as the authors also proved that bromine and peroxidasin interact in vivo to strengthen collagen IV scaffolds [12]. Thus, as tissue development is conserved throughout the animal kingdom, the requirement of bromine for collagen synthesis also applies to humans [12,21].

Despite all genetic and physiological conservation, the *D. melanogaster* model is limited in the study of some aspects of TE physiology, as some biochemical pathways evolved only in higher organisms or solely within the vertebrate lineage. For instance, unlike humans, the fruit fly neither expresses orthologs of the iodine-dependent thyroid hormones T3 (tri-iodothyronine) and T4 (thyroxine) nor deiodinases and sodium iodine symporters, while there are still indications for endogenous synthesis and metabolization of iodinated tyrosines in the fly [22]. Selenium is essential for humans, as it is used for the synthesis of 25 known selenoproteins [23]. In contrast, the requirement of Se in fruit flies, which contains only three selenoproteins, has not been confirmed since genetic disruption of selenoprotein biosynthesis has no effect on lifespan [24]. In this regard, it is of note that some insect species lack selenoproteins at all [25]. Among the known transition metals that act as coenzyme and as the central atom of the corrin ring of vitamin B12, cobalt is as likely to be required in insects as it is for methylation reactions in humans. However, studies on the requirements of Co and vitamin B12 in *D. melanogaster* are lacking and should regard B12 supply through the gut microbiota [26]. Although it is poorly absorbed, Cr has been demonstrated to affect insulin resistance in patients suffering from type 2 diabetes mellitus. Indications for a potentially similar role of chromate in the carbohydrate metabolism of the fruit fly are given since chromate appears to also possess some insulin-mimetic properties in the fruit fly [27].

In *D. melanogaster*, the essential metals iron (Fe), copper (Cu), and zinc (Zn) are tied to homeostatic regulation, of which the underlying mechanisms are well described [14,16,19,28]. However, only little is known about the homeostatic mechanisms of other TE in the fruit fly. Such mechanisms may include metallothionines and other TE-binding proteins that initiate accumulation or transport as well as proteins involved in cellular import and export that will be expressed in the respective organs and tissues.

**Figure 1:**
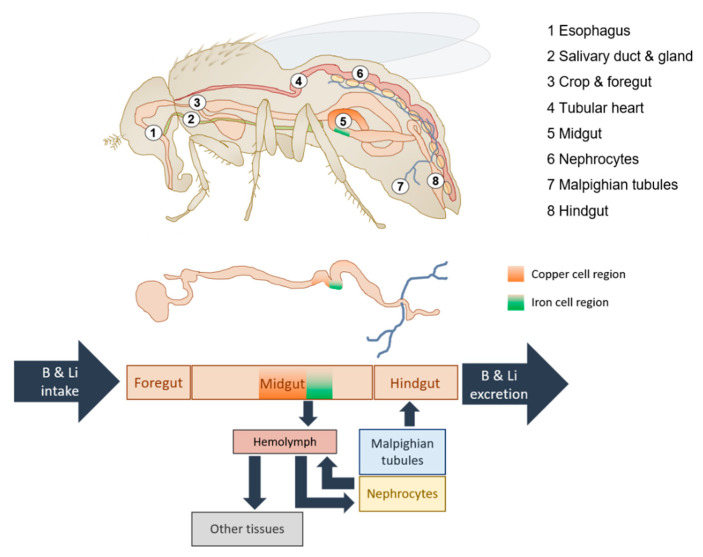
*D. melanogaster* as a versatile model organism studying trace element bioactivity and homeostasis. The upper panel depicts the anatomy of the fruit fly restricted to key organs in the putative homeostatic regulation of trace elements based on Lemaitre et al. (2013) [29], Tatar et al. (2014) [30], and Bilder et al. (2021) [31]. Accordingly, the lower panel depicts a working hypothesis on B and Li homeostasis. Dietary B and Li enter the body most probably in the midgut region (small intestine equivalent) and are then dispersed via the hemolymph (blood equivalent) to the organs/tissues of the fruit fly. Nephrocytes and the Malpighian tubules (kidney equivalents) are the prime candidates for B and Li excretion releasing the trace elements into the hindgut (colon equivalent) for defecation.

A closer look into the fly’s structure of the regulatory organs and their putative homeostatic function is given in Figure 1 [6,29,30,31]. In *D. melanogaster*, the gut is arranged as a simple epithelium subdivided into the foregut, midgut, and hindgut [29]. Being part of the diet, trace elements are consumed orally as the food is liquified and predigested by the saliva that is secreted onto the food [32]. The chyme travels through the relatively impermeable foregut and reaches the midgut where nutrients are region-specifically further digested and absorbed. Depending on the expression of specific transporters, TE, like Fe, Zn, and Cu, are absorbed into the endothelium of the acidic zone of the middle midgut, known as the copper-cell region, followed by the iron-cell region [29]. Once the TE are absorbed into the enterocyte, they are either stored (e.g., Cu bound to metallothionine in copper cells and Fe binding to ferritin in iron cells) or partially released into the circulating hemolymph (bound to complexes or unbound as ions) and thereby transported to other organs and tissues in order to fulfil their bioactive function [6,33,34,35]. The hemolymph is a blood-like, vessel-free fluid that circulates with help of the tubular heart that is located in the abdomen [36]. The mechanism of TE elimination in the fly has not yet been fully elucidated. However, it is a likely assumption that excess amounts are filtered from the hemolymph through the nephrocytes (analogous to the human glomeruli) and discharged through the Malpighian tubules (analogous to renal tubular systems) into the hindgut region for defecation [37,38]. Zn, for example, is accumulated in the Malpighian tubules where importers of extracellular Zn are expressed to initiate disposal of the element [14].

### 1.3. Importance of Choosing the Appropriate D. melanogaster Diet

For all nutrients, the most accepted and most obvious proof of essentiality is a deprivation being lethal to an organism or, at least, resulting in severe health damage. Yet, to provoke signs of deficiency, one has to keep the diet mostly free from the respective element, which is often practically impossible when complex diets are applied. However, for *D. melanogaster,* there is an option of keeping the diet rather defined in its nutritional composition. While complex diets based on yeast, carbohydrate sources, and agar are commonly used for cultivation and treatment of the fruit fly, some groups have introduced semi-defined (meridic) or completely chemically defined (holidic) diets to study certain aspects of physiology [39]. For example, to prove essentiality of bromine in *D. melanogaster*, the authors created a meridic costume diet with undetectable levels of bromine by culturing yeast in a bromine-free media [12]. In the meantime, more elaborated chemically defined fruit fly media have been developed allowing a more standardized dietary treatment [39,40]. Monitoring the real value of the respective element in the fly diet and preferring a chemically defined diet can be highly recommended when working within trace quantities. Nevertheless, it is obvious that such chemically defined diets will be a key tool in the study of potentially essential nutrients.

## 2. Boron (B)

With around 10-ppm abundance in the continental crust, B is a rather rare element on Earth finding its way into our food chain primarily through plant-based foods, such as fruits (e.g., plums, peaches, grapes), fruit juices, wine, nuts, and vegetables, but also through drinking water [41,42,43]. Animal-derived foods (e.g., meat, dairy products, and eggs) and grains contain lower amounts of B [41]. In foods, B naturally occurs as inorganic borates like boric acid (BA) or as organic sugar-borate complexes like calcium fructoborate [44]. Relatively high levels of B were detected in peaches, table wine, peanuts, and raisins ranging from 0.5 to 2.2 mg/100 g [45]. The WHO defines safe ranges of daily intake for adults between 1 and 13 mg, while the average intake settles around 1.2 mg/d via solid food and 0.2–0.6 mg/d through drinking water [8]. Thereby, B is quantitatively categorized as a trace element in the human diet.

B was found to be essential for vascular plants and some species of algae and fungi and exhibits important functions in trout, zebra fish, frogs, and rats, as it is involved in development, in stimulating growth, or as compound of functional molecules [46,47,48,49,50,51,52]. The vital function of B in plants is well defined. In form of BA, it is used for the structure of cell walls where it forms diester bonds between apiosyl residues of two rhamnogalacturonan II (RGII) monomers, which are conserved complex polysaccharides. As borate-complexed dimers, these structures provide sufficient stability to the cell-wall matrix [46]. Further, B was found to be required for development, growth, pollination, and seed formation in plants [53].

**Figure 2:**
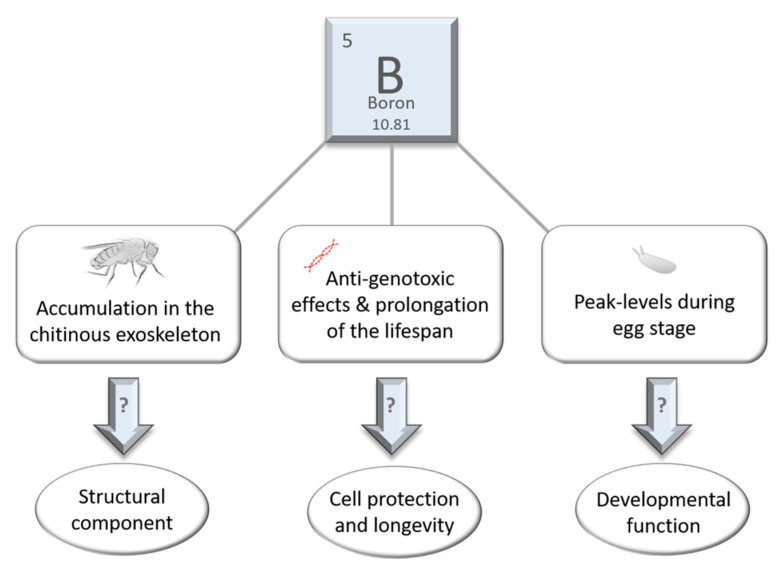
Boron-related bioactivity in *D. melanogaster*. Boron (atomic number: 5, relative atomic weight: 10.81) was found to accumulate in the chitinous exoskeleton of the adult fly indicating that B might be required for structure and stability. Anti-genotoxic effects and prolongation of the lifespan through B-application show that trace amounts of the element may be required for cell health and longevity. Peak levels of B at the egg stage indicate a potentially important function for embryonic development.

While there is no evidence for essentiality of B in humans so far, multiple suggestions are given on beneficial effects of B regarding bone health, wound healing, steroid metabolism, and immune and brain function or affecting the activity of anti-oxidative enzymes as well as mineral absorption [54]. Furthermore, urinary B excretion was negatively correlated with homocysteine and inflammatory parameters in kidney transplant patients [43]. Whether any of these observations rely on one or more specific vital functions of B in humans, or if those effects are simply of non-essential nature has yet to be determined. Likewise, it is unknown if B is essential for the fruit fly. Either way, in nature, *D. melanogaster* is very likely to be exposed to a diet rich in B, as it feeds from yeast on deteriorating fruits and vegetables [55]. The current knowledge of B-related bioactivity in *D. melanogaster* is illustrated in Figure 2 and further discussed in the following.

### 2.1. Homeostatic Regulation of B

In order to perform a vital function in an organism, there has to be a significant amount of the element present in the body even though, in terms of TE, this amount is expected to be comparably low. The greatest amounts would be expected to be accumulated in tissues where the regarded element is required to perform a specific function. In mammals, B appears to accumulate in bones, hair, and nails rather than in soft tissues or fluids [56]. While B levels of human soft tissues, urine, and blood generally range from 0.05 mg/kg to 10 mg/kg, bones contain at around 60 mg/kg [56,57]. Similarly, the adult *D. melanogaster* predominantly accumulates B in the chitinous exoskeleton. Flies that were fed a diet with B-rich mineral water stored 60% (males) or up to 80% (females) of the element in the insoluble fraction of the whole-body homogenate, which mostly consists of the exoskeleton [42]. In line with this, the total body B contents are nearly twice as high in untreated *D. melanogaster* males compared to female flies, the latter exhibiting higher proportions of soft tissue [42,52].

In adult *D. melanogaster*, the whole-body B status can be significantly increased by feeding a B-rich diet. These results are in line with effects of B-rich mineral water on serum B levels in humans [42]. In fruit flies, the B status depends on the developmental stage and the age of the animal. Accordingly, the life-cycle-aligned B status is almost U-shaped, with the highest concentration found during egg stage, declining during larval stages up until adulthood, and again rising with age [58].

While there are data on the distribution of B in *D. melanogaster*, the knowledge of underlying mechanisms relating to its absorption and elimination in the fly are rather scarce. Bioavailability and absorption mechanisms of chemical elements usually depend on how they are presented to the organism, namely as organic or inorganic compounds. In mammals, sodium tetraborate (also referred to as borax) is rapidly and with an assumed bioavailability of 100% absorbed from the gastrointestinal tract to be presented to the blood mostly as non-dissociated BA. Studies on how B absorption and homeostasis may be regulated in *D. melanogaster* are currently lacking. Since BA is absorbed in low pH environments, it will most likely be primarily taken up in the acidic midgut region where metal TE (Fe, Cu, Zn) are thought to be absorbed [29,59].

In mammals, excess B is eliminated through renal excretion. Since we know little about B-elimination in the fly, we can only speculate that a surplus of B may find its way through the equivalent *D. melanogaster* excretory system, which contains the nephrocytes and Malpighian tubules [38]. Future studies should track different B compounds through the organism of the fly in order to support any suggestions on homeostatic regulation of B.

B uptake and distribution in plants is initiated via AtBor1 (*Arabidopsis thaliana* BOR1). The mammalian homolog is NaBC1 (Novel amplified in breast cancer 1) representing a ubiquitous Na^+^-dependent tetrahydroborate transporter [60]. NaBC1 expression is sensitive to B supplementation in mammalian cells. Further, NaBC1 appears to be selective for borate, as it does not transport other metalloids, such as arsenate, and prefers borate over OH^−^. In vitro, borate affects growth-related pathways, such as the mitogen-activated protein kinase (MAPK) pathway, which promotes cell proliferation and growth at higher and suppresses both at lower concentrations [61]. mRNA levels in jejunal epithelia were increased accompanied by a decrease in renal expression in response to 50 mg B/kg diet of growing pigs [62]. In fact, the above-listed findings on NaBC1 may have given prove for a homeostatic regulation and the molecular background on a potential function of B for growth in mammals. Indications for B-binding molecules or B-specific membrane transporter were also found in the human leukemia cell line HL60 where intracellular B was retained against a concentration gradient [63]. Whether or not these findings also account for *D. melanogaster* has yet to be confirmed. At least, a BLAST search using the mammalian NaBC1 amino acid sequence as query did not reveal a clear homolog in the fruit fly genome. The two hits, the annotated *Drosophila* proteins AE2 (anion exchange protein), and Ndae1 (Na^+^-driven anion exchanger 1) exhibit considerably higher degrees of similarity to mammalian bicarbonate transporters SLC4A2 (the sodium-independent bicarbonate/chloride anion-exchanger protein AE2) and SLC4A7 (the sodium bicarbonate co-transporter NBCn1/NBC3). In the African clawed frog *Xenopus laevis*, B is of central importance for embryo-larval organogenesis development. Signs of B-deficiency include morphological abnormalities and a general decrease of embryonic survival [51]. The lipid bilayer of *Xenopus* oocytes is permeable for B to pass via simple passive diffusion, which ensures B supply during development [64].

### 2.2. Beneficial Effects of B on Lifespan

In 1990, Massie et al. pointed out that media, which are commonly used for *Drosophila* treatment in aging studies, vary in their B contents and that the choice of the media substantially affects the outcome of the lifespan experiments. It was found that adding 1, 5, or 10 mM sodium borate to an instant medium containing basal levels of 5.55 mg/kg B (equivalent to 0.5 mM B) decreased the average lifespan dose-dependently by around 20 up to nearly 70%. In contrast, supplementing trace amounts of sodium borate leading to a final B concentration of 11.3 mg/kg slightly but significantly increased the lifespan by 3%. This effect was even enhanced to 9.5% when the B concentration of the control medium was minimized by exchanging conventional yellow corn meal by a white corn meal leading to 0.62 mg/kg B (equivalent to 0.057 mM B) [58]. According to this, supplementing trace amounts of B increases the lifespan in *D. melanogaster*, whereas adding B to a level of 1 mM and above results in a significant decrease in lifespan.

### 2.3. Protective Effects of B on Genotoxic Stressors

Antigenotoxic effects of dietary B were evaluated by applying the recognized somatic mutation and recombination test (SMART) in *D. melanogaster* [65]. The SMART wing spot assay was first introduced by Graf et al. in 1984 and is still used to assess genetic damage induction in somatic cells during larval chemical exposure. Genotoxicity is calculated from wing spots in the adult fly indicating mutagenesis [66]. Hereby, third-instar larvae trans-heterozygous for the two genetic markers *multiple wing hairs* (*mwh*) and *flare* (*flr3*) were fed until pupation with B concentrations ranging from 0.1 to 40 mg/mL diet, co-administrated with 0.1 mM ethyl methansulfonate (EMS), which is a DNA-reactive mutagen and carcinogen causing random point mutations. The treatment was negatively controlled with distilled water or solely 0.1 mM EMS as positive control. At 20 and 40 mg/L B, frequency of clone formation was reduced in the marker heterozygous (MH)-wing flies and in all balancer heterozygous (BH)-wing flies. Co-treatment with B decreased the frequency of total *mwh* spots on MH wings from 0.55 to 0.44 and in BH wings from 0.38 to 0.33. When comparing *mwh* spot frequencies of B groups to the respective B + EMS groups, no significant difference was found. Despite the protective effect of B on EMS-induced genotoxicity, co-treatment did not rescue the EMS-induced reduction in survival rate at any concentration. Taken together, it was found that B diminishes the genotoxic effects of EMS at all concentrations used. The authors suggest that these results could be explained with the repeatedly described antioxidative capacities of B [65].

### 2.4. Adverse Effects and Toxicity of B

For *Drosophila*, most of the currently available B-related data focusses on potential adverse effects and toxicity of relatively high intake levels of B. Although B is not categorized as a toxic trace element by the WHO, and safe upper limits of daily B exposure were not examined, extremely high doses will still negatively affect an organism’s health, just as for any substance [8]. However, evaluating the toxic effects through overdosage could also reveal targets of action and indicate possible physiological functions of the respective element.

In general, the likelihood of B poisoning for humans through dietary intake is remote, and clinical symptoms are only noted at comparably high, single doses above 100 mg [67]. In rats however, the Lowest Observed Adverse Effect Level (LOAEL) of BA is determined at 76 mg/kg/d, which would equal a total of 5.3 g BA per day for a 70 kg average person [68]. Based on human poisoning cases, the lethal dose for adults in form of BA was estimated at 15 to 20 g [69]. Major chronic toxicities of boronated compounds in rodents involve the reproductive system and development, such as, for instance, the impairment of ovulation and spermiation and fetal malformations [70].

In *D. melanogaster*, transgenerational effects of B on sexual differentiation and the number of progenies were monitored (F1 to F5) using a medium that was prepared with water containing either 6 or 12 mg/L B (0.55 and 1.1 mM). The control diet was prepared with distilled water estimated to contain residues of 0.2 to 0.5 mg/L B. The survival rates of all five generations revealed that water B levels of 6 mg/L significantly decreased the lifespan of F1 up to F3 and for 12 mg/L up to F4 before recovering in F4 or F5, respectively. The gender distribution was also significantly affected by the B treatment in both groups. There was a significant increase of male offspring over females in F1, with 55% at 6 mg/L and 60% at 12 mg/L. In F2 to F4, however, the distribution significantly changed in favor of females before recovering again in F5 to an even gender distribution. In addition, a gender-specific effect of B was observed, however, solely in the F3 generation. Here, the percentage of animals with increased body size was significantly higher in males when compared with corresponding female flies. However, this study did not control food intake of the animals, so lifespan effects could be biased by differences in feeding behavior. There is also no information on how the size of the adult flies was determined. The authors suggested that B promotes the expression of the X chromosome, which might affect courtship and mating behavior [71].

Working within much higher ranges of concentration, Erdem et al. studied the toxic effects of 10, 30, 150, 300, or 400 mg/L sodium tetraborate on lifespan and fecundity in the fruit fly. At 400 mg/L, a significant reduction in longevity was found for females and males. Reproductive toxicity of B was already visible at 10 mg/L sodium tetraborate, captured through a reduced number of eggs per female and day [72]. It would be of great interest to elucidate the cause for the reduced oviposition in the fruit fly since reproductive and developmental toxicity of B have been reported in several other species [52,70,73]. Notably, disodium octaborate tetrahydrate, which is applied in pest control of the spotted-wing *Drosophila suzukii*, has delayed actions that are probably caused by adverse effects on fecundity and filial generations [74]. Besides limiting reproduction in *D. suzukii*, BA is also an effective insecticide against the common bed bug, as it destructs the lining of its foregut and triggers signs of neurotoxicity, effects that could be reproducible in the fruit fly [75]. An actual molecular target of B-toxicity is viewed in yeast. According to Aryanitis et al., B-containing antifungals inhibit yeast growth by suppressing protein synthesis. As fungistatic effects of BA are alleviated with ribose, nicotinamide adenine dinucleotide (NAD), and tryptophan, the authors hypothesized that BA possibly disrupts tryptophan synthesis and the downstream co-enzyme NAD [76].

In rats, B tends to accumulate in the testes [77]. High doses of BA can induce testicular atrophy and loss of fertility [78]. Testicular toxicity in response to B was also found in mice and dogs [73,79]. However, workers of the high-exposure group from a BA production plant in Turkey presented blood B concentrations that were rather low compared to serum concentrations reached in animal experiments. Apparently, there was no adverse effects on reproductive toxicity indicators, such as sperm count, motion, and morphology as well as blood levels of luteinizing and follicle-stimulating hormones [80]. Similar results were gathered from high-exposure groups in China showing no signs of altered semen characteristics [81]. According to this, the risk for men to suffer adverse effects on reproductive capacity even under high environmental exposure appears to be rather low.

In wild-type *w^1118^ D. melanogaster*, developmental effects of BA (10–300 mg/L) on the whole-body protein profile were captured by sodium-dodecyl sulfate polyacrylamide gel electrophoresis (SDS-PAGE). Hereby, the protein pattern of three developmental stages (third instar larvae, pupae, and adult flies) were compared. At pupation, 300 mg/L BA caused a strongly visible decline of total proteins when compared to the control and all other treatment groups. In adult males and females, the whole-body protein content increased with the level of BA exhibiting a maximum at 200 mg/L, followed by a severe decline of total protein in adults that reared on 300 mg/L. The authors explain the observed changes in total protein levels as a response to cell damage caused by the excess BA [82]. With this study, the doses of BA causing adverse effects on development up until adulthood start at a concentration of 300 mg/L.

The impact of B on development should be further assessed in *D. melanogaster*, especially since we know that the B status naturally declines from egg stage up until pupation [58]. Capturing effects of excess B during larval development could provide insight into its bioactivity and possible physiological functions in the larvae. In-vitro cultures of two-cell rat embryos in media containing very low (0.006 mM) up to high levels (10 mM) of BA revealed that proper embryonic differentiation and proliferation was observed solely above 2 mM BA. Maternal exposure to a low B diet, however, was associated with degeneration as well as a reduction in blastocyst formation and number [52]. Hereby, indications for a possible vital function of B during development of higher organisms are given and supported through a rather low teratogenicity of the element.

## 3. Lithium (Li)

The highly reactive alkali metal Li has the lowest density and is also the lightest of all solid elements [83]. With an abundance of 20 ppm, there is twice as much Li present in the Earth’s crust compared to B [84].

The highest resources of Li are located in South America, within the so-called Lithium Triangle (Argentina, Bolivia, and Chile), where the alkali metal is progressively mined from salt lakes in order to meet the increasing global demand for batteries and electrical devices [85]. The more Li is lifted to the surface, the more likely it could steadily find its way into our food chain making the evaluation of its physiological impact on us rather urgent. Depending on the region, foods that mainly contribute to the Li supply in humans are grains and vegetables [86]. Higher Li status in humans was also associated with higher intakes of potatoes, leafy vegetables, and root vegetables in the general population [87]. Several mineral and medicinal waters but also tap water may represent a significant source of dietary Li intake [88]. However, it needs to be considered that there may be substantial regional variations in the Li concentration of tap and mineral water that mainly depend on geological factors. Unlike mineral and medicinal waters, most wine and beer, soft and energy drink, and tea and coffee samples were rather Li-poor food items and thus may only contribute to a moderate extent to the dietary Li supply [88]. Furthermore, it has been shown that Li levels of mushrooms can be substantially increased via Li biofortification [89]. Li is often referred to as an ultra-TE (UTE < 1 µg/g of the diet) [90]. In fact, the average daily intake of Li from foods and beverages usually adds up above 650 µg [91]. Some authors recommend a provisional dietary Li intake of approximately 1000 µg/d, which can already be exceeded by drinking a liter of Li-rich drinking water [88,92]. As mentioned before, *D. melanogaster* feeds on yeast on fermenting fruits as their naturally occurring food source. Therefore, the Li supply from its natural diet will most likely depend on the Li content of the ground water, which generally varies between geographic regions [88,93]. Under laboratory conditions, the basal entry of Li from ingredients of the fly diet should be monitored as well as the Li content of the water used.

Li is known for its role in the treatment of bipolar disorder (BD). BD is generally characterized by repetitive manic and depressive episodes, and to this day, Li salts are still the first choice for mood stabilization [94]. Furthermore, Li shows neuroprotective effects in patients suffering from Huntington’s disease, as it prevents neuronal apoptosis, and microdoses of only 300 µg Li per day were reported to stabilize cognitive impairment in patients suffering from Alzheimer’s disease [95,96]. While most of the beneficial clinical effects of Li were attributed to its ability to inactivate GSK-3 (glycogen synthase kinase-3, located downstream of the insulin and Wnt signaling pathway), the exact molecular actions of Li remain largely unknown [97]. Apart from its medical application, there is also evidence that low-Li drinking water is correlated with higher suicidal rates as well as drug use, crimes, and acts of violence in some countries [91,98]. Taken together, these findings suggest that Li may support mental health and cognitive function in humans.

### 3.1. Homeostatic Regulation of Li

Since Li has been used as a drug for decades, there is quite a great deal of information regarding the absorption, distribution, and elimination of the element in animal models and humans. Like B, Li is rapidly absorbed by the gastrointestinal tract with high bioavailability [86,88]. Li from inorganic compounds, such as carbonate and citrate, will be presented to the gut as ionized Li^+^, which is thought to be absorbed into the enterocytes via sodium channels [86]. While Li is almost evenly distributed throughout the aqueous phase of the human body, levels of the cerebrospinal fluid are only 40% of the plasma levels [99]. As for sodium, Li retention takes place in the proximal tubule [100]. With increasing dietary supply, human serum levels and urinary Li excretion rise in a dose-dependent manner [88]. However, to maintain any possible vital functioning of Li, there may only be a considerably low status required. Whereas in humans, the Li status is thought to be tied to the homeostatic mechanisms of sodium, little is known about Li regulation in the fly. In *D. melanogaster*, sodium uptake takes place in the midgut and hindgut region [29]. Likely candidates for Li uptake in the fly may comprise the solute carrier (SLC) superfamily, which includes passive transporters and ion-coupled transporters and exchangers [101]. Na^+^-reabsorption occurs in the hindgut region, while its elimination is managed by the energy-demanding ion secretion of the Malpighian tubules [102]. It is likely to assume that Li regulation is also tied to the fruit fly’s homeostatic mechanisms of sodium as it was described in mammals.

In wild-type *w^1118^ Drosophila* fed a standard diet, the whole-body Li content was detected at concentrations around 12.5 µg/kg for males and around 11 µg/kg for females. When providing a diet with Li-rich mineral water, the element’s status significantly increased in male and female flies, whereby it appears to stay slightly higher in males compared to females at any concentration given [88].

### 3.2. Beneficial Effects of Li on Lifespan

**Figure 3:**
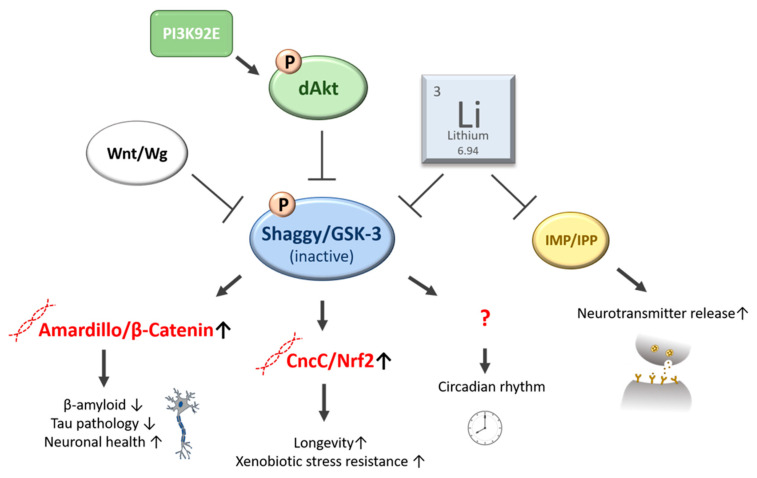
Overview of molecular targets of lithium in *D. melanogaster*. In the fly, lithium (atomic number: 3, relative atomic weight: 6.94) is thought to inhibit two major targets: (i) Shaggy/GSK-3, which is also a target of the insulin signaling and the Wnt pathway, and (ii) IMP/IPP. In disease models of *D. melanogaster*, inactivation/phosphorylation of Shaggy/GSK-3 increases intracellular levels of the transcriptional factor Amardillo/β-Catenin, which is associated with decreasing levels of beta-amyloid and tau pathology as well as improvement of neuronal health. Li-induced inactivation of Shaggy can also increase levels of the transcription factor CncC/Nrf2, which is likely to be the cause of the observed longevity and xenobiotic stress resistance. Effects of Li on the circadian rhythm are not yet explained but are also associated with Shaggy-inhibition. Independently of Shaggy/GSK-3, Li increases neuronal signaling, as it promotes synaptic neurotransmitter vesicle release through inhibition of IMP/IPP. PI3K, phosphatidylinositol 3-kinase; dAkt, protein kinase B; Wnt/Wg, wingless; Shaggy/GSK-3, glycogen synthase kinase 3; IMP/IPP, Inositol monophosphatase/Inositol polyphosphate 1-phosphatase; CncC/Nrf, cap-n-collar C/Nuclear factor E2-related factor 2.

When tested in the context of aging, two general health promoting effects of Li were observed in the *D. melanogaster* model: (1) an extension of the life- and health-span independently of sex and genetic background and (2) the resistance to xenobiotic stressors [103]. It was found that Li supplementation (1–25 mM LiCl) can enhance longevity in *Drosophila* even if only administered mid-life or as short-term treatment. Further, Li supplementation mitigated the age-related decline in locomotor activity indicative for an improved health-span. As mentioned above, the most captured effect of Li at the molecular level is the inhibition of GSK-3. In line with this, the observed lifespan extension in *D. melanogaster* is most likely induced by inactivation of Shaggy (*sgg*), the fruit fly GSK-3 functional homolog, as inhibitory phosphorylation of Shaggy was significantly increased by Li treatment. In addition, the reduced lifespan caused by ubiquitous overexpression of GSK-3 was partially rescued by feeding 10 or 25 mM LiCl. Remarkably, Shaggy/GSK-3 is also phosphorylated and thereby inactivated through the protein kinase B/Akt, which is part of the evolutionary, highly conserved insulin/IGF-1 signaling (IIS) pathway (see Figure 3) [103]. In various models, alteration of the IIS affect the health and lifespan of the respective organism [104,105].

When further unraveling the underlying molecular mechanisms of Li supplementation, the Shaggy/GSK-3 downstream target Cap’n’Collar C (CncC, the fly homolog to Nrf-2) needs to be considered. Inactive phosphorylated Shaggy is thought to increase intracellular levels of the transcriptional factor CncC/Nrf-2, which in turn regulates numerous genes crucially involved in the detoxification of xenobiotics and reactive oxygen species. In *D. melanogaster*, the transcriptional response of Li treatment overlaps with that induced by *cncC* overexpression. In good accordance, the Li-treated flies showed an increased resistance towards xenobiotic stressors phenobarbital and paraquat [103]. Most importantly, stimulated CncC/Nrf-2 has been frequently reported to be associated with the prevention of age-related diseases and longevity [106].

Interestingly, Li also significantly extends the lifespan of *D. melanogaster* females when fed a high-sugar diet containing 200 g/L sucrose and blocked the high-sugar induced increase of triglycerides at 1 mM LiCl [103]. Li therefore appears to promote longevity regardless of the time of administration, avert sugar-induced alterations of the lipid metabolism in the fly, and to improve resistance to xenobiotic stressors (see Figure 3).

An increase of lifespan due to dietary Li was also reported for other models, such as *Caenorhabditis elegans* and fission yeast [107,108]. Recently, Li was also found to mildly increase the health status of mice at old age regarding glucose tolerance, body weight, and fat storage as well as locomotor function and kidney health. However, despite these effects, Li supplementation did not lead to a prolonged lifespan in these mice [109].

### 3.3. Li affects the Circadian Rhythm

*D. melanogaster* has been versatilely used to study health promoting effects, the impact on behavior, and the therapeutic value of Li-salts in the treatment of neurological pathologies. In patients suffering from mental illness, such as BD or depression, the biological rhythm of the sleep-wake cycle is often disrupted [110]. For example, in BD adults, the so-called evening type is quite commonly described [110,111]. In order to study effects of Li on the circadian system of the fruit fly, locomotor activity was captured at 0.3–300 mM LiCl showing that the period of the circadian rhythm is lengthened and arrhythmicity increased through Li treatment [112]. Apparently, an increased length of the circadian period was also observed in *D. melanogaster sgg* loss-of-function mutants, while in humans, the manifestation of BD correlates with a single nucleotide polymorphism in the GSK-3β promoter region (see Figure 3) [113,114]. Hence, human and fly appear to respond similarly to Li treatment in regards of the circadian rhythm.

### 3.4. Li in the Treatment of Neurodegeneration and Cognitive Impairment

Over the years, the interest in Li has grown towards its medical efficacy in the treatment of neurodegenerative diseases, such as Alzheimer’s, Huntington’s, and Parkinson‘s disease [115]. In order to gain a better understanding of the molecular targets of Li in neuronal health, effects of Li treatment were studied in a number of neurodegenerative disease models in *D. melanogaster*. Alzheimer’s disease (AD) is caused by the deposition of pathogenic proteins in the brain, called β-amyloid plaques and tau fibrils [116]. As these structures accumulate, neuronal communication is increasingly impaired, which progressively results in synaptic loss, cell death, and dementia [117]. Likewise, in the adult-onset *Drosophila* model of AD, β-amyloid accumulation increases mortality and neuronal dysfunction, which can in fact be rescued by chronic Li treatment, as endogenous fly tau protein but not transcript levels were reduced [118]. Apparently, Li treatment in the AD *Drosophila*-model reduces overall protein synthesis and thereby also ß-amyloid production, which results in a rescue of AD-induced locomotory defects at 10 mM LiCl and a rescue of the lifespan reduction at 10 to 50 mM LiCl. It was also found that through the inhibitory actions of Li on GSK-3, symptoms of tau pathology can be reversed in the fly [119]. In AD, the canonical Wnt/β-catenin signaling pathway, which affects major cellular processes, such as neuronal survival and neurogenesis, is drastically downregulated [120]. Migrating β-amyloids elevate intracellular GSK-3, which in turn inhibits the expression of proneural genes as the required transcription factor β-catenin is inactivated and degraded [121]. Li treatment of *D. melanogaster* S2 cells was reported to mimic the Wg (Wingless) signaling and to induce accumulation of the β-catenin encoding homolog Amardillo, most likely through inhibition of GSK-3 [105]. Intracellular Amardillo/β-catenin accumulation through Li may promote neuronal health and prevent neurodegeneration (see Figure 3).

Another neurodegenerative pathology, which is related to alterations of the Wnt/β-catenin signaling, is Huntington’s disease (HD). HD is an autosomal dominant disease caused by a CAG expansion within the coding region of the *huntingtin* gene. HD symptoms are assigned to the consequential polyglutamine-mediated toxicity [122]. In the transgenic *Drosophila* disease model, HD is characterized by an adult-onset rhabdomere loss in the fly’s eyes, which allows quantification of neurodegeneration. Li-treatment (4.2 mM or 0.18 g/L) significantly attenuates photoreceptor loss and improves survival rates in polyglutamine-mediated toxicity in the HD model, which is again related to Shaggy/GSK-3-inhibition and thus activation of Wg/Amardillo signaling [123].

Li was also found to rescue phenotypes in models of some lesser known genetically-caused neuropathological disorders. Spinocerebellar ataxia type 3 (SCA3) is an autosomal dominant neurodegenerative disorder, which comes along with severe loss of motor coordination and peripheral neuropathy. In the transgenic SCA3 *Drosophila* model, chronic Li treatment prevented eye depigmentation, relieved locomotor disability (climbing ability), and increased the median lifespan at a dose of 5–15 mM LiCl [124]. Li has also been tested in the treatment of age-dependent cognitive impairment using the fragile X model of *D. melanogaster* [125]. In humans, X-chromosomal mental retardation varies from mild learning disabilities to severe cognitive impairment [126]. In the fruit fly model, a change in copulation behavior as well as an age-related decline in learning capacity and short-term memory were described, caused by enhanced metabotropic glutamate receptor signaling. All of these phenotypes were rescued by Li administration of 5 mM LiCl suggesting yet another Li-responsive neurological disorder [127]. A recently published article revealed that Li may also be affective in the treatment of the Cornelia de Lange syndrome (CdLS). CdLS is characterized by a delay in neurodevelopment leading to morphological abnormalities of the brain structure. In the CdLS model of *D. melanogaster*, an abnormal mushroom body morphology is caused by a loss of function allele (*Nipped*-B407). Li treatment (100 mM LiCl) of the parental generation resulted in a rescue of the mushroom body morphology in their adult offspring. Further, the authors described that therapeutic effects of Li on CdLS may be associated with Wnt signaling [127].

Apart from Wnt and GSK-3, the mammalian inositol monophosphatase (IMP), orthologous to inositol polyphosphate 1-phosphatase (IPP) in the fruit fly, is also a putative molecular target that is strongly associated with Li-induced effects on neuronal function [128]. IPP/IMP is a Mg^2+^-dependent, Li-sensitive enzyme involved in the regulation of the inositol metabolism limiting the availability of free inositol and thus regulating synaptic transmission. Hyperexcitable *D. melanogaster ipp* mutants display defects of synaptic transmission due to a severe increase of vesicle release in larval neuromuscular junctions. This synaptic defect is phenocopied in wild-type Canton-S flies by acute Li exposure, while *ipp* mutants are resistant to Li treatment [129]. Accordingly, it is suggested that Li inhibits IPP in the fruit fly, whereby synaptic vesicle release is increased, which promotes neuronal signal transduction (see Figure 3).

Lastly, at rather high doses (25–100 mM LiCl and 200–250 mM Li citrate), Li salts were found to relieve the seizure-like mutant *Shudderer* from its characteristic sporadic leg jerks, which cause the fly to shudder and lurch [130,131]. This phenotype is caused by a gain of function mutation in the *Drosophila* voltage-gated sodium channel gene *paralytic* (*para*). Li-salts are thought to counteract the increased sodium channel activation in this seizure-like hyperexcitability mutant [132]. However, the underlying mechanisms are unsolved.

### 3.5. Adverse Effects and Toxicity of Li

The European Chemical Agency (ECHA) calculated the No Observed Adverse Effect Level (NOAEL) of Li carbonate from human long-term treatment data revealing that no adverse effects are expected up to a daily oral intake of 1.2 mg/kg body weight [133]. Overall, Li toxicity in mammals usually targets intrauterine development, the kidneys, and thyroid. Common diseases that are associated with the pharmacological application of Li are nephrogenic diabetes insipidus, hypothyroidism and goiter, as well as fetal cardiac malformation, especially the Ebstein’s anomaly [100,133,134].

In *D. melanogaster*, two studies were conducted focusing on developmental toxicity of Li in the fly. Back in 1923, Mann was the first to study effects of oral Li application in *D. melanogaster* by adding 0.03 or 0.05% of Li_2_CO_3_ (300 or 500 mg/L diet, equivalent to 4 or 6.77 mM) to banana agar. It was shown that Li delayed development in the fruit fly at both concentrations [135]. Thirty years later, King tested the developmental toxicity of five salts including LiCl, which apparently turned out to be the most toxic in the wild-type strain Canton. The sex ratio was not affected by 10 mM LiCl, and at 20 mM LiCl, there was already a severe delay of larval and pupal development, which is why the majority of F1 could not be evaluated for gender distribution. However, the ones counted revealed a drastic shift of the ratio towards males, of which most had missing, deformed, or rotated reproductive organs. King explains the morphogenetic abnormalities with the observed ability of Li to increase the density of cytoplasm, which may affect the rotation of the posterior part in males during pupation [136].

GSK-3 activity is strongly associated with growth-mediating effects [137]. Accordingly, it is very likely to expect that drug-induced inhibition of the kinase led to adverse effect on development. In mammalian embryonic stem cells, GSK-3 is involved in cell differentiation, proliferation, and self-renewal [138,139]. Interfering with such sensitive processes by applying high concentrations of Li may be the cause for a delay of development in *D. melanogaster*.

Although it is also one of the proposed molecular targets of Li in *D. melanogaster*, inhibition of IPP/IMP may not be the cause of teratogenic effects in the fruit fly. *Drosophila ipp* mutants are viable and show compensatory upregulation of an alternative branch that ensures inositol availability. Yet, as mentioned above, *ipp* mutants show defects in synaptic transmission due to high vesicle release at larval muscular junctions, phenocopied by Li in wild-type synapses [125]. Vertebrates, however, display negative effects of Li on vasculogenesis and dorsoventral specification, which is prevented with myo-inositol pre-treatment [140].

In a more recent study, the role of a putative amino acid transporter of the solute carrier 6 (SLC6) family was evaluated regarding resistance to Li toxicity. Expression of this transporter called Lithium-inducible SLC6 transporter (*List*) was significantly upregulated in the heads and bodies of virgin female Canton-S flies in response to a treatment of 50 mM LiCl. In mutant *List-da*-KD-1 flies, this upregulation was not found, but instead *List* KD flies became uncoordinated and suffered from impaired climbing behavior in response to the high Li treatment, while the wild-type strain was not affected. Furthermore, mortality rates of *List* KD flies were significantly higher than in Canton-S controls during the Li treatment. Hence, this transporter was identified as a novel Li-specific molecular player that contributes to the resistance of Li toxicity. In that regard, it is of note that cell-type-specific RNAi experiments revealed that flies become most sensitive towards Li toxicity when *List* expression was suppressed in glia cells (compared to *List*-specific gene silencing in neurons or muscles) [141]. *List* is described to hold transporter activities for L-amino acids and neurotransmitters, and its expression can apparently be altered by Li application [142,143]. As mentioned above, in dietary and pharmacological doses, Li is discussed to contribute to the mental wellbeing and cognition Therefore, this study may be important in regards of a potential biological Li-specific function in the fruit fly.

## 4. Conclusions

As outlined in this review, diet composition comes into focus when the fruit fly model is employed to unravel the function of trace metals, such as Li or B. First, since base levels of chemical elements may vary among the different fly diets, and one needs to work within small, defined quantities, it is highly recommended to capture the element’s basal level in the medium. Second, when intending to study essentiality of an element, the most obvious proof is its depletion, which should result in severe health damage or death. While there are no data available on effects of complete withdrawal on neither B nor Li in the fly (restricted to the fact that base entry of the diet has to be zero) to date, no definite claims can be made in regards of their essentiality. Third, supplementation studies are appropriate to elucidate the bioavailability as well as beneficial effects and toxicity of trace elements. Accordingly, studies with fruit fly diets enriched in Li and B revealed that the status of both elements can be elevated in the fruit fly by concentrations that are in the range of natural dietary intake levels. However, to date, little is known about the mechanisms that regulate the B and Li status in flies. Similar to human bones, data indicate that the fruit fly accumulates most of the available dietary B in the chitinous exoskeleton, which might suggest an important role of B in the formation of the skeletal structure. Moreover, peak levels at the egg stage of *Drosophila* along with a relatively low teratogenicity may point to a function of B during the development of the fruit fly, which warrants further investigation. Several supplementation studies carried out in the *D. melanogaster* model provide indications on vital functions and comparability to the situation in humans for both elements. In many cases, the underlying cellular and molecular mechanisms in the context of health, ageing, and disease prevention have yet to be fully understood. This is especially true for B, whereas the majority of the Li-induced effects can be ascribed to GSK3/Shaggy inhibition in both mammals and fruit flies. Overall, consistent results of flies and mammals in respect to distribution, toxicity, and biological activity show that *D. melanogaster* can be an adequate and versatile model organism in the study of trace elements that hold potentially essential functions.
